# Toxic heavy metal concentrations in multiple sclerosis patients: A systematic review and meta-analysis

**DOI:** 10.17179/excli2021-3484

**Published:** 2021-11-19

**Authors:** Sorour Sarihi, Maryam Niknam, Sanaz Mahjour, Mahnaz Hosseini-Bensenjan, Fatemeh Moazzen, Sahar Soltanabadi, Hamed Akbari

**Affiliations:** 1 Shiraz HIV/AIDS Research Center, Institute of Health, Shiraz University of Medical Sciences, Shiraz, Iran; 2 Department of Biochemistry, School of Medicine, Shiraz University of Medical Sciences, Shiraz, Iran; 3 Department of Psychiatry and Behavioral Sciences Psychiatry, Northwestern University, Feinberg School of Medicine, USA; 4 Hematology Research Center, Shiraz University of Medical Sciences, Shiraz, Iran; 5 Department of Hematology, Faculty of Allied Medicine, Bushehr University of Medical Sciences, Bushehr, Iran; 6 Student Research Committee, Shiraz University of Medical Sciences, Shiraz, Iran; 7 Department of Biochemistry, School of Medicine, Kerman University of Medical Sciences, Kerman, Iran; 8 Student Research Committee, School of Medicine, Kerman University of Medical Sciences, Kerman, Iran

**Keywords:** heavy metal, multiple sclerosis, meta-analysis, arsenic, cadmium

## Abstract

The present meta-analysis was performed to assess the association between MS patients and control subjects in terms of their circulating levels of arsenic (As), lead (Pb), mercury (Hg), and cadmium (Cd). We searched Medline/PubMed, Scopus, Web of Science, and Embase up until June 2020 to identify all studies that examined the concentrations of heavy metals in MS patients. Statistical tests used to assess inter-study heterogeneity were Cochrane's Q test and the I^2^ statistic. Given the observed significant heterogeneity, the random-effects model was employed to pool the weighted mean differences (WMDs) and the corresponding 95 % confidence intervals (CIs). Out of a total of 1181 articles, 16 studies with 1650 participants (772 patients with MS and 878 controls) were included in this review meta-analysis. Pooled results using random-effects model showed that the levels of Pb (WMD= 0.73 µg/L, 95 % CI= 0.33 to 1.12, P< 0.001), As (WMD= 2.48 μg/L, 95 % CI= 1.44 to 3.53, P <0.001; I^2^= 98.9 %, P <0.001), and Cd (WMD= 0.17 μg/L, 95 % CI= 0.09 to 0.26, P <0.001) were significantly higher in MS patients than those of the controls. However, there were no significant differences in the levels of Hg (WMD= -0.14 µg/L, 95 % CI= -0.77 to 0.49, P= 0.658) among both groups. Sensitivity analysis indicated that after excluding one-by-one study, the overall pooled WMD of Pb was changed. This meta-analysis showed that patients with MS had significantly higher levels of circulatory As and Cd compared to the controls. Yet, there was no statistically significant difference between circulating levels of Hg and Pb among MS patients and controls. See also Figure 1(Fig. 1).

## Introduction

Characterized by an immune-mediated disease caused by an autoimmune attack on the central nervous system (CNS) and the progressive demyelination through chronic inflammation, multiple sclerosis (MS) is one of the most common neurological diseases (Dehghanifiroozabadi et al., 2019[12]; Nirooei et al., 2021[36]). The onset and progression of MS have been attributed to irreversible degeneration of myelinated axon accompanied by the lower capability for remyelination in the early stages and plaques formation as a result of the chronicity in later stages (Cariccio et al., 2019[8]). This disease is the major cause of disability in young and middle-aged people in developed countries (Kotelnikova et al., 2017[27]). Yearly, MS affects more than 2.5 million people globally and the majority of patients have a relapsing-remitting type of disease (Giacoppo et al., 2014[17]; Nicoletti et al., 2016[35]). The exact etiology of MS is not yet known, however several factors including immunological, genetic, environmental and infectious ones, as well as, dietary, habits and life-styles contribute to its occurrence and progression (Forte et al., 2005[15]; Juybari et al., 2018[23]). 

Out of various proposed environmental factors, heavy metals may play a role in MS pathogenesis (Monnet-Tschudi et al., 2006[33]). Given the high toxicity and long-time persistency of heavy metals in the ecosystem, they have the most detrimental effect on human health among the different environmental pollutions including exposure to sunlight, bacteria, viruses, and xenobiotics such as chemicals, drugs, and metals (Aliomrani et al., 2017[3]; Juybari et al., 2018[23]). 

These toxicants exert their toxicity through various mechanisms, including free radical formation, cell membrane disruption, enzyme inhibition, and induction of autoimmunity (Juybari et al., 2018[23]). Furthermore, studies have shown that exposure to these neurotoxicants intensifies the progression of MS (Siblerud and Kienholz, 1994[44]; Attar et al., 2012[5]; Pachner, 2012[37]; Ghoreishi et al., 2015[16]; Kahrizi et al., 2016[24]; Razavi et al., 2016[41]). 

As nonessential heavy metals, arsenic (As), lead (Pb), mercury (Hg), and cadmium (Cd) are xenobiotics with toxic effects on public health (Papastergios et al., 2004[39]; Saeedi et al., 2014[43]). As environmental factor, Pb plays an important role in the etiopathogenesis of MS through a multi-action process, including the production of free radical species, deactivation of antioxidant sulfhydryl pools, inhibition of enzyme activity, blocking the physiological absorption of essential trace elements, and induction of auto-antibodies production against myelin proteins (Aliomrani et al., 2016[2]; Hayatbakhsh et al., 2017[18]; Dehghanifiroozabadi et al., 2019[12]). Another significant neurotoxin with a possible role in the pathogenesis of MS is Hg which is involved in the inhibition of myelin production and decreases the nerve conduction velocity (Forte et al., 2005[15]; Attar et al., 2012[5]). Furthermore, with the highest toxicities in the human neurological system, As may trigger the development or progression of MS by inducing the production of reactive oxygen species (ROS) in neuronal cells, mitochondrial dysfunction, inflammation, and hyperphosphorylation of the tau protein, all of which lead to axon injury in MS (Ratnaike, 2003[40]; Dangleben et al., 2013[11]). It has been proposed that Cd toxicity can cause severe damage to organs such as brain, testis, kidney, lung and liver. Moreover, based on animal experimental studies, Cd induces neurological abnormalities, cerebral hemorrhage, and neonatal cerebral edema. It has been revealed that Cd enhances the formation of reactive radicals and interferes with antioxidant enzymes activity in adult rat brain by alteration of membrane-bound enzymes such as Na+/K+ ATPase and structural lipids integrity (Aliomrani et al., 2016[2]). 

With regard to the global nature of the heavy metals' contamination, precisely determining the associations between these environmental contaminants and MS is crucial for knowing the etiology of the disease and for informing the public health attempts to reduce exposure to toxic metals. The existing systematic review and meta-analysis was designed to summarize the available data regarding the circulating levels of Pb, Hg, As, and Cd with potential mechanistic associations with MS, between MS patients and control subjects.

## Methods

A systematical search of online databases, i.e. Medline/PubMed, Scopus, Web of Science (ISI), and Embase was performed up until June 2020 using the combination of MeSh terms and keywords: ["multiple sclerosis (MS)"]2 AND ["heavy metal poisoning" OR "metal poisoning" OR "heavy metal" OR "heavy metals" OR "toxic heavy metal" OR "toxic metal" OR "metal toxicity" OR "arsenic (As)" OR "cadmium (Cd)" OR "mercury (Hg) "OR "lead (Pb)"]. The reference lists were manually checked in the pertinent studies and previous peer-reviews to find additional related articles. Our literature searches were limited to studies published in the English language.

### Study selection

Two investigators (MH-B and FM) independently screened the literature search results using EndNote X8 by reviewing article titles and abstracts to find the pertinent studies in accordance with the inclusion criteria. Discrepancies in this process were resolved through discussion with a third author (Sahar Soltanabadi).

The eligibility inclusion criteria were: articles were originally observational studies (cross-sectional, case-control, or cohort), encompassed patients with MS, studies that compared the heavy metal concentrations in patients with MS and controls, and studies that reported or were able to calculate the mean changes of heavy metal concentrations including Hg, Pb, As, and Cd, along with their standard deviations (SDs) for both MS patients and control group. Meanwhile, studies that did not provide sufficient information on mean±SD changes of heavy metal concentrations for MS patients and controls, the abstracts without full papers, animal studies, *in vitro*, letters, editorials, case report or series, randomized controlled trials, reviews, and studies that did not have a comparison/control group were excluded.

### Data extraction

Three individual authors (HA, Sorour Sarihi, and MH-B) extracted all required data using pre-designed data collection sheets in Excel. The extracted data included: author's name, publication year, basic demographic characteristics, the study design and location, sample size (patients with MS and controls), type of MS, the technique of measurement for heavy metal levels, and the mean and SD for Pb, Hg, As, and Cd in patients with MS and controls.

### Quality assessment

The methodological quality of the included studies was examined using the Newcastle-Ottawa Scale (NOS) according to the following three points: "the participants' selection procedure, comparability of included groups, and outcome/exposure ascertainment". Based on this tool, the results for each cohort or case-control design with scores ≥ 7 and for cross-sectional design with scores ≥ 5 were considered as a high-quality study (see Supplementary Table 1).

### Data synthesis and statistical analysis 

A comprehensive search in the literature (through manual and online electronic tools) was performed to avoid any publication bias. Additionally, we used statistical Begg's rank-correlation (Begg and Berlin, 1989[6]) and Egger's regression asymmetry (Egger et al., 1997[14]) to evaluate the evidence of potential publication bias across the included studies in the current meta-analysis. Inter-study heterogeneity was identified using Chi-square and I^2^ statistics. An I^2^ greater than 50 % and P < 0.1 for Chi-square test represented a significant inter-study heterogeneity (Higgins and Thompson, 2002[19]). The changes of heavy metal concentrations between MS patients and the control group were estimated through the calculation of the weighted mean difference (WMD) and the corresponding 95 % confidence interval (CI) using STATA software version 11.0 (STATA Corp, College Station, Texas). Given the wide variety of indications among included studies to pool SMDs, random-effects models were employed to conduct meta-analyses. Further analyses including Subgroup and univariate meta-regression analyses were conducted to assess the source of heterogeneity based on categorical and continuous variables, respectively. Meanwhile, a sensitivity analysis was administered to investigate the impact of each study on the reliability of the pooled WMD. P-values less than 0.05 were considered as statistically significant.

## Results

**Figure 2:**
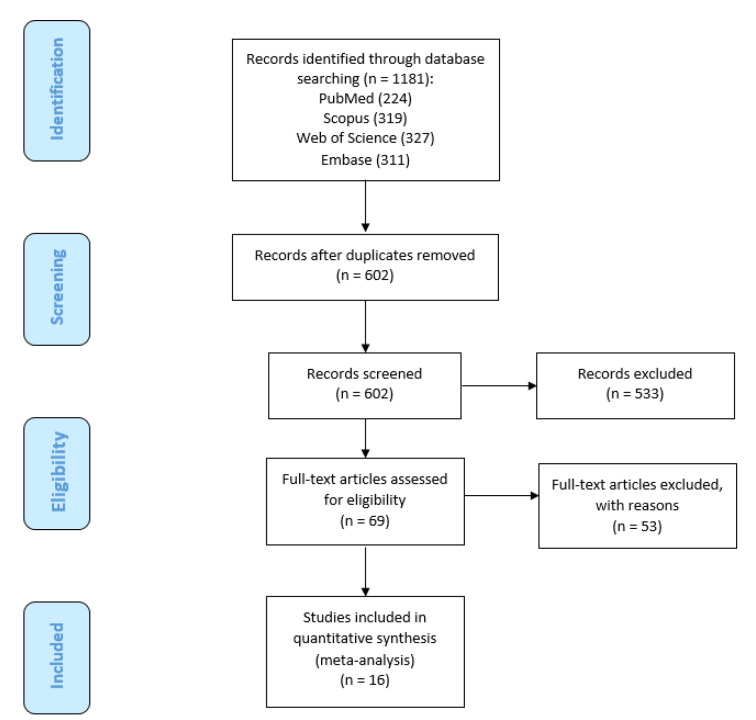
The PRISMA flowchart of study identification and selection process

The flowchart for the systematic searches in the literature and the step-by-step study identification process are summarized in Figure 2(Fig. 2). Overall, 16 articles (Janghorbani et al., 2017[20]; Forte et al., 2005[15]; Visconti et al., 2005[46]; Alimonti et al., 2007[1]; Madeddu et al., 2011[30]; Ristori et al., 2011[42]; Attar et al., 2012[5]; Giacoppo et al., 2014[17]; Yousefi et al., 2014[48]; Ghoreishi et al., 2015[16]; Aliomrani et al., 2016[2]; Aliomrani et al., 2017[3]; Juybari et al., 2018[23]; Dehghanifiroozabadi et al., 2019[12]; Paknejad et al., 2019[38]; Nashmi et al., 2020[34]) out of 1181 records were identified through literature searching, and then selected to be eligible for the current meta-analysis regarding our criteria for inclusion. These articles included a total of 1650 participants (772 patients with MS and 878 controls). Only one study was of a cohort design (Ristori et al., 2011[42]), all other fifteen studies (see above) were of a case-control design. Thirteen studies have reported on Pb (Janghorbani et al., 2017[20]; Forte et al., 2005[15]; Visconti et al., 2005[46]; Alimonti et al., 2007[1]; Madeddu et al., 2011[30]; Ristori et al., 2011[42]; Giacoppo et al., 2014[17]; Ghoreishi et al., 2015[16]; Aliomrani et al., 2016[2]; Aliomrani et al., 2017[3]; Dehghanifiroozabadi et al., 2019[12]; Paknejad et al., 2019[38]; Nashmi et al., 2020[34]), eleven on Cd (Janghorbani et al., 2017[20]; Forte et al., 2005[15]; Visconti et al., 2005[46]; Alimonti et al., 2007[1]; Madeddu et al., 2011[30]; Ristori et al., 2011[42]; Ghoreishi et al., 2015[16]; Aliomrani et al., 2016[2]; Aliomrani et al., 2017[3]; Paknejad et al., 2019[38]; Nashmi et al., 2020[34]), six on Hg (Forte et al., 2005[15]; Visconti et al., 2005[46]; Alimonti et al., 2007[1]; Ristori et al., 2011[42]; Attar et al., 2012[5]; Giacoppo et al., 2014[17]), four on As levels (Yousefi et al., 2014[48]; Aliomrani et al., 2017[3]; Juybari et al., 2018[23]; Paknejad et al., 2019[38]) in patients with MS and controls. Studies were published between 2005 and 2020. Nine of the included studies were conducted in Iran (Janghorbani et al., 2017[20]; Attar et al., 2012[5]; Yousefi et al., 2014[48]; Ghoreishi et al., 2015[16]; Aliomrani et al., 2016[2]; Aliomrani et al., 2017[3]; Juybari et al., 2018[23]; Dehghanifiroozabadi et al., 2019[12]; Paknejad et al., 2019[38]), six in Italy (Forte et al., 2005[15]; Visconti et al., 2005[46]; Alimonti et al., 2007[1]; Madeddu et al., 2011[30]; Ristori et al., 2011[42]; Giacoppo et al., 2014[17]), and one in Iraq (Nashmi et al., 2020[34]). Details of the basic characteristics of the included studies are presented in Table 1(Tab. 1) (References in Table 1: Alimonti et al., 2007[1]; Aliomrani et al., 2016[2], 2017[3]; Attar et al., 2012[5]; Dehghanifiroozabadi et al., 2019[12]; Forte et al., 2005[15]; Ghoreishi et al., 2015[16]; Giacoppo et al., 2014[17]; Janghorbani et al., 2017[20]; Juybari et al., 2018[23]; Madeddu et al., 2011[30]; Nashmi et al., 2020[34]; Paknejad et al., 2019[38]; Ristori et al., 2011[42]; Visconti et al., 2005[46]; Yousefi et al., 2014[48]).

### Meta-analysis

#### Mercury

**Figure 3:**
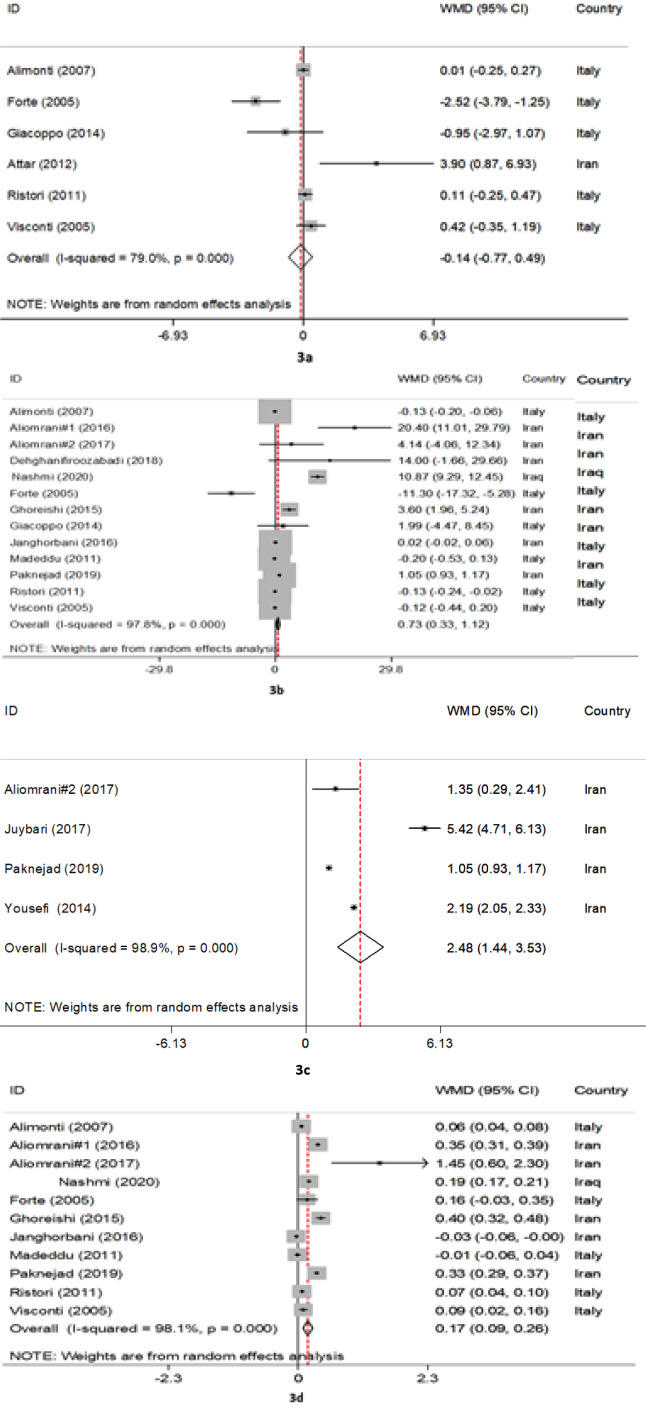
The forest plots of pooled estimates of the weighted mean differences on mercury (a), lead (b), arsenic (c), and cadmium (d) between MS patients and control groups

The pooled results obtained on the basis of six included articles using the random-effects model revealed that Hg levels (WMD= -0.14 µg/L, 95 % CI= -0.77 to 0.49, P= 0.658) in MS patients were not statistically different from those of the controls (Figure 3a(Fig. 3); References in Figure 3: Alimonti et al., 2007[1]; Aliomrani et al., 2016[2], 2017;[3] Attar et al., 2012[5]; Dehghanifiroozabadi et al., 2019[12]; Forte et al., 2005[15]; Ghoreishi et al., 2015[16]; Giacoppo et al., 2014[17]; Janghorbani et al., 2017[20]; Juybari et al., 2018[23]; Madeddu et al., 2011[30]; Nashmi et al., 2020[34]; Paknejad et al., 2019[38]; Ristori et al., 2011[42]; Visconti et al., 2005[46]; Yousefi et al., 2014[48]). The evidence of significant heterogeneity across the included articles (I^2^=79.0 %, P < 0.001) showed that the subgroup analyses were conducted according to the country (Italy vs. Iran vs. Iraq) and the type of MS (relapsing-remitting MS (RR-MS) vs. unspecified MS vs. other). As indicated in Table 2(Tab. 2), we found that the mercury levels were not different across diverse countries and types of MS. Furthermore, meta-regression analyses explicated that total sample size (β= 0.01, P= 0.833), publication year (β= 0.17, P= 0.507), and study quality score (β= 0.21, P= 0.913) had no statistically significant impact on mercury levels. In addition, sensitivity analysis showed that the exclusion of each study from the meta-analysis did not change the pooled WMD (see Supplementary Figure 1). Statistical publication bias results showed there was no evidence of publication bias (P for Begg's test= 0.573 and for Egger's test= 0.843).

#### Lead 

Thirteen articles comprised of a total of 1326 subjects have investigated the Pb concentrations. The pooled results using a random-effects model indicated a significant difference in the Pb levels between MS patients and controls (WMD= 0.73 µg/L, 95 % CI= 0.33 to 1.12, P< 0.001) (Figure 3b(Fig. 3)). According to significant inter-study heterogeneity (I^2^= 97.8 %, P< 0.001), additional analyses were carried out. It was found that studies conducted in Iran significantly differed in terms of Pb levels (WMD= 1.39 µg/L, 95 % CI= 0.44 to 2.34, P= 0.004) as well as in patients with other types of MS (WMD= 0.63 µg/L, 95 % CI= 0.18 to 1.09, P= 0.007) vs. other strata (Table 2(Tab. 2)). The results of meta-regression analyses revealed that continuous factors as total sample size (β= -0.01, P= 0.771) and study quality score (β= -1.31, P= 0.756) did not have any significant effects on Pb concentrations, but the publication year was positively related to Pb levels (β= 0.77, P= 0.024). Furthermore, sensitivity analysis indicated that after excluding Diame Nashmi's study (Nashmi et al., 2020[34].) from our meta-analysis, the overall WMD was changed (see Supplementary Figure 2). There was no publication bias as to the use of quantitative analyses as Begg's (P= 0.143) and Egger's tests (P= 0.223).

#### Arsenic 

Four studies comprised of 219 patients with MS and 2396 controls have reported As levels. The meta-analysis results using random-effects model showed that arsenic levels were significantly increased in patients with MS compared with controls (WMD= 2.48 μg/L, 95 % CI= 1.44 to 3.53, P <0.001; I^2^= 98.9 %, P <0.001) (Figure 3c(Fig. 3)). Despite the stratification of included studies based on country and type of MS, we found no significant changes in pooled WMD in different subgroups (Table 2(Tab. 2)). Meta-regression analysis indicated that the total sample size (β= -0.03, P= 0.507), publication year (β= -0.15, P= 0.840), and study quality score (β= 2.49, P= 0.088) had no significant impact on the association between MS patients and the levels of arsenic. Additionally, sensitive analysis results exhibited that the exclusion of Yousefi's study ( 2014[48].) changed the pooled WMD (see Supplementary Figure 3). Begg's test (P= 0.996) and Egger's test (P= 0.512) showed no significant publication bias, quantitatively.

#### Cadmium

The pooled results from 11 included studies showed that levels of Cd among patients with MS were significantly higher than those of controls (WMD= 0.17 μg/L, 95 % CI= 0.09 to 0.26, P <0.001) (Figure 3d(Fig. 3)). Statistical tests for heterogeneity were significant across the included studies (I^2^= 98.1 %, P <0.001). Subgroup analysis based on the type of MS presented significantly increased Cd levels in patients with RR-MS (WMD= 0.35 μg/L, 95 % CI= 0.28 to 0.42) and with unspecified MS (WMD= 0.18 μg/L, 95 % CI= 0.02 to 0.34) vs. other strata (Table 2(Tab. 2)). Also, the results of meta-regression analyses found that factors such as total sample size (β= 0.01, P= 0.641), publication year (β= 0.02, P= 0.235), and study quality score (β= 0.00, P= 0.641) were associated with Cd levels. The findings of sensitive analysis demonstrated reliable pooled WMD after excluding the studies one by one (see Supplementary Figure 4). The evidence of publication bias was not quantitatively observed across included studies although Begg's test (P= 0.586) and Egger's test (P= 0.397) were employed.

## Discussion

Not only is MS a disabling disease but it is also the leading cause of acquired neurological disability in young adults (Carvalho, 2013[9]; Ebrahimi-Kalan et al., 2014[13]). Even though focal inflammatory plaques and axonal loss are found to be the main pathological features of MS; however, the precise etiology of the disease is yet unknown (Loma and Heyman, 2011[29]; Koch et al., 2013[26]). Recently, it has been suggested that toxic heavy metals as environmental factors play a significant role in MS pathogenesis. Furthermore, a strong association has been shown between exposure to heavy metals and a higher incidence of MS (Ghoreishi et al., 2015[16]). To the best of the authors' knowledge, this is the first systematic review and meta-analysis that examined the associations between circulating levels of Pb, Hg, As, and Cd with MS. The World Health Organization (WHO) has listed these heavy metals among ten chemicals with a major concern for public health (WHO, 2020[47]). It is worth noticing that apart from one study performed in Iraq, other included studies in this meta-analysis were conducted in Iran and Italy as countries with increasing in MS prevalence rate. Accordingly, the findings obtained from the present study demonstrated that the circulating levels of As and Cd were significantly higher in patients with MS than in the healthy controls. Nevertheless, there was no statistically significant difference between circulating levels of Hg among MS patients and controls. Although Pb level was significantly higher in patients with MS compared to the healthy controls, after sensitivity analysis and removing one study (Nashmi et al., 2020[34]), Pb level was insignificantly different between the two groups.

One of the most toxic naturally occurring metals, As is a peripheral neurotoxicant capable of inducing nervous system damages and neuropathy (Yousefi et al., 2014[48]; Alizadeh-Ghodsi et al., 2018[4]). This toxicant can induce or intensify many diseases including MS by producing oxidative stress (Jomova et al., 2011[21]). The present study revealed that As had significantly higher circulating levels among patients with MS than in the healthy controls. Yet, high heterogeneity was also seen. Subgroup analyses revealed that studies related to As neurotoxicity were carried out in Iran (Yousefi et al., 2014[48]; Aliomrani et al., 2017[3]; Juybari et al., 2018[23]; Paknejad et al., 2019[38]) out of which, one study (Yousefi et al., 2014[48]) showed that As levels were significantly higher in undetermined MS subgroup than other types. It has been reported that in Tabriz, as the third most polluted city of Iran, excessive concentration of arsenic from industrial pollutions increases oxidative stress levels in MS patients (Yousefi et al., 2014[48]). Meanwhile, further studies on a larger population are required to confirm this hypothesis. 

As a redox inert element, Cd is one of the most poisonous heavy metals with an extreme health hazard to humans and animals (Ghoreishi et al., 2015[16]; Aliomrani et al., 2016[2]). It has been suggested that Cd makes an indirect contribution to increases in the free radical formation and to oxidative stress via Fenton reaction (Méndez-Armenta and Ríos, 2007[32]). Cd-induced neurotoxicity has been connected with neurodegenerative diseases such as MS (Branca et al., 2018[7]). Findings obtained from the present study showed significantly higher circulating levels of Cd in patients with MS compared to the healthy controls. Following the subgroup analyses, the observed heterogeneity was lowered within some of the subgroups, including Italian and RR-MS ones. It is worth noting that Italians showed significantly higher Cd levels in MS patients compared to controls, emphasizing the need for further studies to identify the cause of excess Cd in MS patients in this region. Furthermore, Cd levels were significantly higher in RR-MS subgroups compared with other types. It is highly suggested that more cohort and clinical trial studies be conducted to assess the impact of Cd chelation therapy in RR-MS patients, as the most common type of MS. It has been reported that glutathione S-Transferase Mu 1 (GSTM1) null genotype is likely to be responsible for susceptibility to Cd toxicity in RR-MS patients, particularly in patients with a smoking habit, therefore, we advise that future studies should consider the genotype feature of the patient to define the exact reason of higher Cd levels in RR-MS patients (Aliomrani et al., 2017[3]). 

One of the most toxic heavy metals, Hg has acute and chronic toxic effects on the human body including the nervous and immune systems (Kim and Zoh, 2012[25]; Kahrizi et al., 2016[24]). An important neurotoxicant, Hg is involved in the elimination of the myelin sheath and inflammatory status progression of MS (Forte et al., 2005[15]; Attar et al., 2012[5]; Cariccio et al., 2019[8]). Based on the present study, though MS patients showed a slightly lower Hg level compared to the control group, no statistically significant difference was found in Hg levels between MS patients and controls. The two forms of Hg that can be absorbed by an organism causing toxicity are methylmercury and mercury vapor (Clarkson and Magos, 2006[10]; Magos and Clarkson, 2006[31]). It is strongly suggested that subsequent studies should measure these chemical forms of Hg in MS patients.

Pb is a redox-active and broadly dispersed metal (Aliomrani et al., 2016[2]; Dehghanifiroozabadi et al., 2019[12]). As a very toxic and persistent metal, Pb is a major risk to human health (Kumar and Scott Clark, 2009[28]; Jomova and Valko, 2011[22]; Tabrizi et al., 2021[45]). Pb can serve as a hapten through binding to myelin proteins and it is considered that Pb is responsible for the production of auto-antibodies against myelin proteins, thereby playing a crucial role in MS pathogenesis (Razavi et al., 2016[41]). With respect to Pb, a significant difference was found between the two groups in the present meta-analysis. However, after excluding one study by Nashmi et al. (2020[34]), the heterogeneity was reduced and Pb circulating levels were not significantly different after conducting the sensitivity analysis. Therefore, additional larger studies are required to clarify the exact role of this toxic metal in the pathogenesis of MS. Pb subgroup analyses also showed the significantly higher Pb levels in Iranian MS patients compared to controls, though the heterogeneity remained high. In Iran, cities such as Tehran and Isfahan have a high prevalence of MS. In this regard, exposure to absorbable Pb in air-suspended particles and soil as the main sources of Pb in Tehran and Isfahan, respectively, has been shown to be significantly linked with MS prevalence (Aliomrani et al., 2016[2]; Dehghanifiroozabadi et al., 2019[12]). Additional studies are needed to discover other possible reasons which could account for Pb excess in patients suffering from MS in these regions.

The strengths and limitations of this meta-analysis require careful attention. This is the first comprehensive meta-analysis addressing a number of major environmental toxic metals in relation to MS. A subgroup analysis was led to ascertain the possible sources of heterogeneity in our study. Statistical publication bias results showed there was no evidence of publication bias. Our meta-analysis has also some limitations which are as follows. First, studies included in this meta-analysis are heterogeneous. Thus, the results of this study should be interpreted with caution. Second, a small number of studies was included in this meta-analysis and additional large scale studies are required to confirm our conclusions. Third, age and sex are very significant variables in studies regarding the influence of metals metabolism on MS. It is an aspect of this study that was not considered in this study because the included articles lacked individual participant data. Furthermore, this meta-analysis did not take account of the confounding factors, including chemical forms, times, doses, and routes of exposure to heavy metals, dietary habits, lifestyles, and smoking habits, all of which influence the neurotoxic effects of these toxicants. Additionally, the identification of polymorphisms that impact circulating levels of these toxic metals, such as polymorphisms in GST, may also pave the way for future experiments on potential causal associations of these metals with MS. Ultimately, most studies included in the meta-analysis were conducted on MS patients from Iran and Italy. It is yet uncertain whether the findings could be applied to other populations.

## Conclusion

Findings demonstrated that higher levels of As and Cd were associated with MS, however, no significant differences were found in terms of Hg and Pb. Given the high heterogeneity among the included studies, the results should be interpreted with caution. Besides, further large scale clinical studies are required to investigate the role of toxic metals in the development of MS. Considering the great socio-economic burden of MS on patients and families, particularly, in Italy and Iran, in the future, it is necessary to take measures to decrease human exposure to these environmental toxicants and possibly reduce the risk of MS development. 

## Declaration

### Ethics approval and consent to participate

Not applicable.

### Consent for publication

Not applicable.

### Availability of data and materials

The dataset analyzed during the current study are available from the corresponding author on reasonable request.

### Competing interests

The authors declare that they have no conflict of interest.

### Funding

None.

### Authors' contributions

All named authors met the International Committee of Medical Journal Editors (ICMJE) criteria for authorship for this article, take responsibility for the integrity of the work as a whole, and have given their approval for this version to be published.

### Acknowledgments

Not applicable.

## Supplementary Material

Supplementary information

## Figures and Tables

**Table 1:**
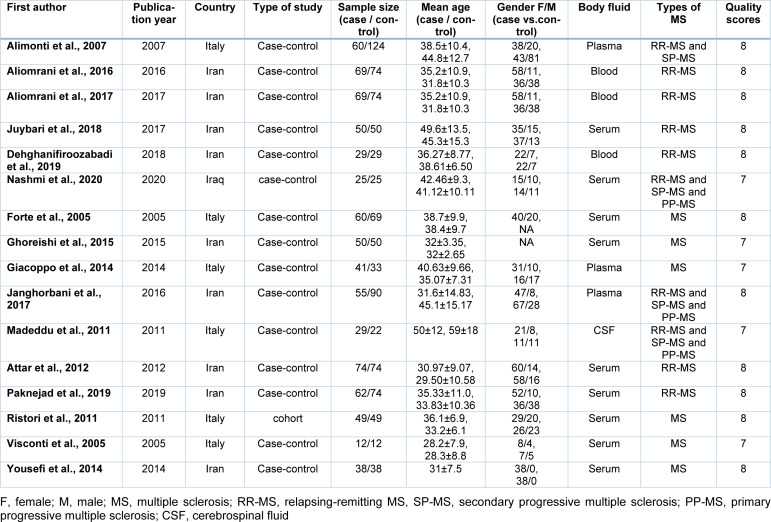
Main characteristics of included studies

**Table 2:**
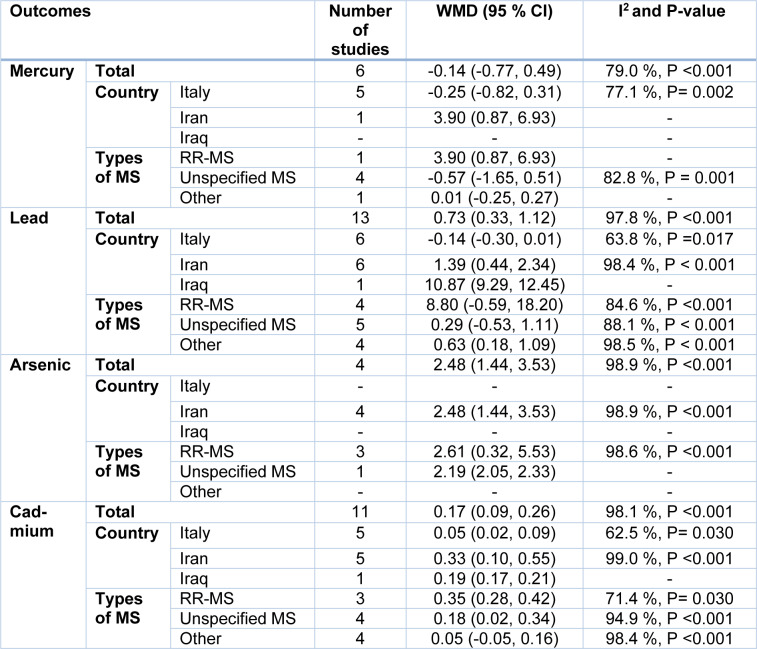
Findigs of subgroup analyses

**Figure 1:**
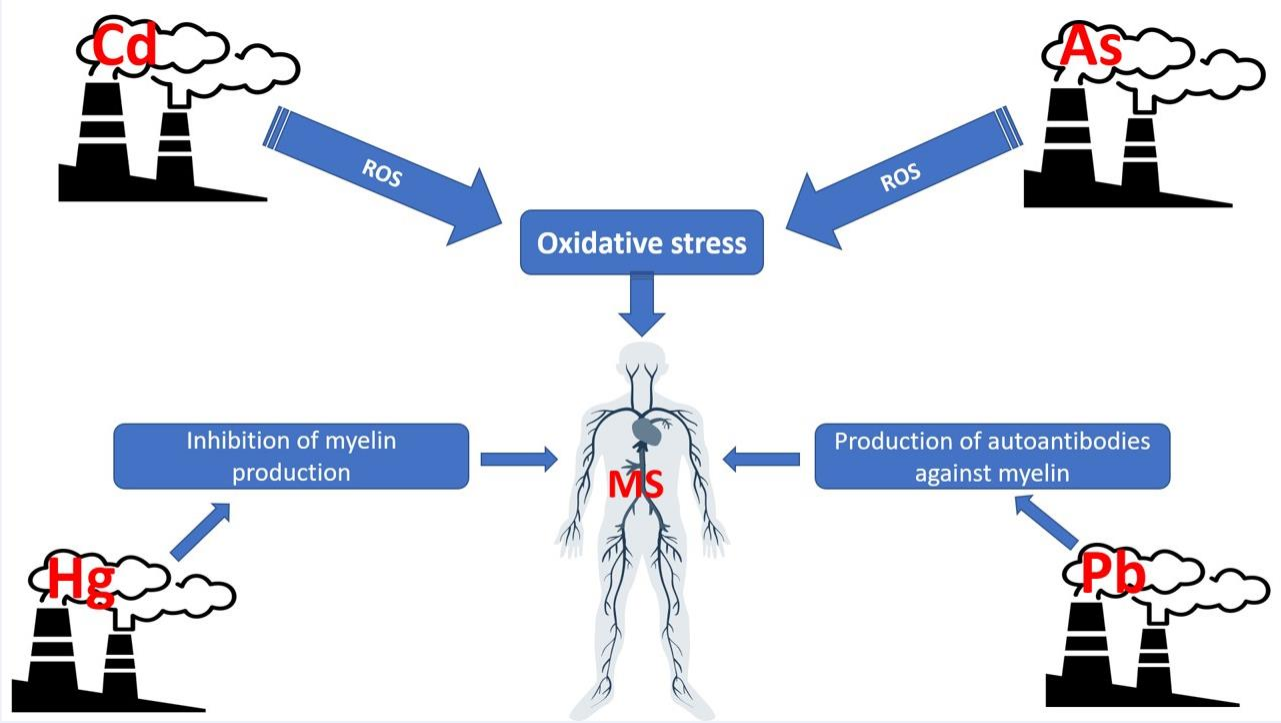
Graphical abstract

## References

[1] Alimonti A, Ristori G, Giubilei F, Stazi MA, Pino A, Visconti A (2007). Serum chemical elements and oxidative status in Alzheimer's disease, Parkinson disease and multiple sclerosis. Neurotoxicology.

[2] Aliomrani M, Sahraian MA, Shirkhanloo H, Sharifzadeh M, Khoshayand MR, Ghahremani MH (2016). Blood concentrations of cadmium and lead in multiple sclerosis patients from Iran. Iran J Pharm Res.

[3] Aliomrani M, Sahraian MA, Shirkhanloo H, Sharifzadeh M, Khoshayand MR, Ghahremani MH (2017). Correlation between heavy metal exposure and GSTM1 polymorphism in Iranian multiple sclerosis patients. Neurolog Sci.

[4] Alizadeh-Ghodsi M, Zavvari A, Ebrahimi-Kalan A, Shiri-Shahsavar MR, Yousefi B (2018). The hypothetical roles of arsenic in multiple sclerosis by induction of inflammation and aggregation of tau protein: a commentary. Nutr Neurosci.

[5] Attar AM, Kharkhaneh A, Etemadifar M, Keyhanian K, Davoudi V, Saadatnia M (2012). Serum mercury level and multiple sclerosis. Biol Trace Elem Res.

[6] Begg CB, Berlin JA (1989). Publication bias and dissemination of clinical research. J Natl Cancer Inst.

[7] Branca JJV, Morucci G, Pacini A (2018). Cadmium-induced neurotoxicity: still much ado. Neural Regen Res.

[8] Cariccio VL, Samà A, Bramanti P, Mazzon E (2019). Mercury involvement in neuronal damage and in neurodegenerative diseases. Biol Trace Elem Res.

[9] Carvalho KS (2013). Mitochondrial dysfunction in demyelinating diseases. Semin Pediatr Neurol.

[10] Clarkson TW, Magos L (2006). The toxicology of mercury and its chemical compounds. Crit Rev Toxicol.

[11] Dangleben NL, Skibola CF, Smith MT (2013). Arsenic immunotoxicity: a review. Environ Health.

[12] Dehghanifiroozabadi M, Noferesti P, Amirabadizadeh A, Nakhaee S, Aaseth J, Noorbakhsh F (2019). Blood lead levels and multiple sclerosis: A case-control study. Mult Scler Relat Disord.

[13] Ebrahimi-Kalan A, Rad JS, Kafami L, Mohammadnejad D, Roudkenar MH, Khaki AA (2014). MS14 down-regulates lipocalin2 expression in spinal cord tissue in an animal model of multiple sclerosis in female C57BL/6. Iran Biomed J.

[14] Egger M, Davey Smith G, Schneider M, Minder C (1997). Bias in meta-analysis detected by a simple, graphical test. BMJ.

[15] Forte G, Visconti A, Santucci S, Ghazaryan A, Figà-Talamanca L, Cannoni S (2005). Quantification of chemical elements in blood of patients affected by multiple sclerosis. Ann Ist Super Sanita.

[16] Ghoreishi A, Mohseni M, Amraei R, Alizadeh A, Mazloomzadeh S (2015). Investigation the amount of copper, lead, zinc and cadmium levels in serum of Iranian multiple sclerosis patients. J Chem Pharm Sci.

[17] Giacoppo S, Galuppo M, Calabrò RS, D’Aleo G, Marra A, Sessa E (2014). Heavy metals and neurodegenerative diseases: an observational study. Biol Trace Elem Res.

[18] Hayatbakhsh MM, Oghabian Z, Conlon E, Nakhaee S, Amirabadizadeh AR, Zahedi MJ (2017). Lead poisoning among opium users in Iran: an emerging health hazard. Subst Abuse Treat Prev Policy.

[19] Higgins JP, Thompson SG (2002). Quantifying heterogeneity in a meta-analysis. Stat Med.

[20] Janghorbani M, Shaygannejad V, Hakimdavood M, Salari M (2017). Trace elements in serum samples of patients with multiple sclerosis. Athens J Health.

[21] Jomova K, Jenisova Z, Feszterova M, Baros S, Liska J, Hudecova D (2011). Arsenic: toxicity, oxidative stress and human disease. J Appl Toxicol.

[22] Jomova K, Valko M (2011). Advances in metal-induced oxidative stress and human disease. Toxicology.

[23] Juybari KB, Ebrahimi G, Moghaddam MAM, Asadikaram G, Torkzadeh-Mahani M, Akbari M (2018). Evaluation of serum arsenic and its effects on antioxidant alterations in relapsing-remitting multiple sclerosis patients. Mult Scler Relat Disord.

[24] Kahrizi F, Salimi A, Noorbakhsh F, Faizi M, Mehri F, Naserzadeh P (2016). Repeated administration of mercury intensifies brain damage in multiple sclerosis through mitochondrial dysfunction. Iran J Pharm Res.

[25] Kim M-K, Zoh K-D (2012). Fate and transport of mercury in environmental media and human exposure. J Prev Med Publ Health.

[26] Koch MW, Metz LM, Agrawal SM, Yong VW (2013). Environmental factors and their regulation of immunity in multiple sclerosis. J Neurol Sci.

[27] Kotelnikova E, Kiani NA, Abad E, Martinez-Lapiscina EH, Andorra M, Zubizarreta I (2017). Dynamics and heterogeneity of brain damage in multiple sclerosis. PLoS Comput Biol.

[28] Kumar A, Scott Clark C (2009). Lead loadings in household dust in Delhi, India. Indoor Air.

[29] Loma I, Heyman R (2011). Multiple sclerosis: pathogenesis and treatment. Curr Neuropharmacol.

[30] Madeddu R, Forte G, Bocca B, Tolu P, Sotgiu MA, Sotgiu G (2011). Heavy metals and multiple sclerosis in Sardinian population (Italy). Anal Lett.

[31] Magos L, Clarkson TW (2006). Overview of the clinical toxicity of mercury. Ann Clin Biochem.

[32] Méndez-Armenta M, Ríos C (2007). Cadmium neurotoxicity. Environ Toxicol Pharmacol.

[33] Monnet-Tschudi F, Zurich MG, Boschat C, Corbaz A, Honegger P (2006). Involvement of environmental mercury and lead in the etiology of neurodegenerative diseases. Rev Environ Health.

[34] Nashmi AD, Hassan AF, Hammady MM (2020). Estimation the level of metals (lead, cadmium, copper and zinc) in multiple sclerosis patients in Basra\Iraq. Ind J Forensic Med Toxicol.

[35] Nicoletti A, Messina S, Bruno E, Mostile G, Quattrocchi G, Raciti L (2016). Risk factors in multiple sclerosis: a population-based case–control study in Sicily. Background and methods. Neurol Sci.

[36] Nirooei E, Kashani SMA, Owrangi S, Malekpour F, Niknam M, Moazzen F (2021). Blood trace element status in multiple sclerosis: a systematic review and meta-analysis. Biol Trace Elem Res.

[37] Pachner AR (2012). A primer of neuroimmunological disease.

[38] Paknejad B, Shirkhanloo H, Aliomrani M (2019). Is there any relevance between serum heavy metal concentration and BBB leakage in multiple sclerosis patients?. Biol Trace Elem Res.

[39] Papastergios G, Georgakopoulos A, Femândez-Turiel J, Gimeno D, Filippidis A, Kassoli-Fournaraki A (2004). Heavy metals and toxic trace elements contents in soils of selected areas of the Kavala Prefecture, Northern Greece. Bull Geol Soc Greece.

[40] Ratnaike RN (2003). Acute and chronic arsenic toxicity. Postgrad Med J.

[41] Razavi Z, Jokar M, Allafchian A, Hossinpour Z, Berenjani L, Nejad VS (2016). The relationship between blood lead levels and clinical features among multiple sclerosis patients in Isfahan, Iran. Iran J Health Saf Environ.

[42] Ristori G, Brescianini S, Pino A, Visconti A, Vittori D, Coarelli G (2011). Serum elements and oxidative status in clinically isolated syndromes: imbalance and predictivity. Neurology.

[43] Saeedi M, Salmanzadeh M, Jamshidi-Zanjani A, Li L (2014). Response to the comments of Zhang et al.(2014) on "heavy metals and polycyclic aromatic hydrocarbons: pollution and ecological risk assessment in street dust of Tehran". J Hazard Mater.

[44] Siblerud RL, Kienholz E (1994). Evidence that mercury from silver dental fillings may be an etiological factor in multiple sclerosis. Sci Total Environ.

[45] Tabrizi R, Sarihi S, Moazzen F, Hosseini-Bensenjan M, Malekpour F, Asadikaram G (2021). A systematic review and meta-analysis on blood lead level in opium addicts: an emerging health threat. Biol Trace Elem Res.

[46] Visconti A, Cotichini R, Cannoni S, Bocca B, Forte G, Ghazaryan A (2005). Concentration of elements in serum of patients affected by multiple sclerosis with first demyelinating episode: a six-month longitudinal follow-up study. Ann Istit Super Sanita.

[47] WHO, World Health Organization (2020). Ten chemicals of major public health concern. https://www.who.int/news-room/photo-story/photo-story-detail/10-chemicals-of-public-health-concern.

[48] Yousefi B, Ahmadi Y, Ghorbanihaghjo A, Faghfoori Z (2014). Serum arsenic and lipid peroxidation levels in patients with multiple sclerosis. Biol Trace Elem Res.

