# Supplementary material for: Toxic heavy metal concentrations in multiple sclerosis patients: A systematic review and meta-analysis
Source: EXCLI J. 2021 Nov 19;20:1571–84. doi: 10.17179/excli2021-3484 (PMC8678057; doi:10.17179/excli2021-3484)
Supplement: Supplementary information [file EXCLI-20-1571-s-001.pdf]

EXCLI Journal 2021;20:1571-1584 – ISSN 1611-2156
Received: February 05, 2021, accepted: October 18, 2021, published: November 19, 2021

Supplementary information to:

Review article:

TOXIC HEAVY METAL CONCENTRATIONS IN MULTIPLE SCLEROSIS PATIENTS: A SYSTEMATIC REVIEW AND META-ANALYSIS

Sorour Sarihi[1], Maryam Niknam[2], Sanaz Mahjour[3], Mahnaz Hosseini-Bensenjan[4], Fatemeh Moazzen[5], Sahar Soltanabadi[6*], Hamed Akbari[7,8]

[1] Shiraz HIV/AIDS Research Center, Institute of Health, Shiraz University of Medical Sciences, Shiraz, Iran
[2] Department of Biochemistry, School of Medicine, Shiraz University of Medical Sciences, Shiraz, Iran
[3] Department of Psychiatry and Behavioral Sciences Psychiatry, Northwestern University, Feinberg School of Medicine, USA
[4] Hematology Research Center, Shiraz University of Medical Sciences, Shiraz, Iran
[5] Department of Hematology, Faculty of Allied Medicine, Bushehr University of Medical Sciences, Bushehr, Iran
[6] Student Research Committee, Shiraz University of Medical Sciences, Shiraz, Iran
[7] Department of Biochemistry, School of Medicine, Kerman University of Medical Sciences, Kerman, Iran
[8] Student Research Committee, School of Medicine, Kerman University of Medical Sciences, Kerman, Iran

* **Corresponding author:** Sahar Soltanabadi, Student Research Committee, Shiraz University of Medical Sciences, Shiraz, Iran. Tel:+98 917 713 3214;
E-mail: saharsoltany@gmail.com

http://dx.doi.org/10.17179/excli2021-3484

This is an Open Access article distributed under the terms of the Creative Commons Attribution License (http://creativecommons.org/licenses/by/4.0/).

EXCLI Journal 2021;20:1571-1584 – ISSN 1611-2156
Received: February 05, 2021, accepted: October 18, 2021, published: November 19, 2021

Supplementary Table 1: Overall quality scores of including studies using Newcastle-Ottawa Scale (NOS) tool

Author name	Study design	Selec-tion	Compara-bility	Exposure/ outcome	Overall quality assessment score
**Alimonti**	Case-control	****	*	***	8
**Aliomrani, 2016**	Case-control	****	*	***	8
**Aliomrani, 2017**	Case-control	****	*	***	8
**Juybari**	Case-control	****	*	***	8
**Dehghani-firoozabadi**	Case-control	****	*	***	8
**Nashmi**	Case-control	***	*	***	7
**Forte**	Case-control	****	*	***	8
**Ghoreishi**	Case-control	***	*	***	7
**Giacoppo**	Case-control	***	*	***	7
**Janghorbani**	Case-control	****	*	***	8
**Madeddu**	Case-control	***	*	***	7
**Attar**	Case-control	****	*	***	8
**Paknejad**	Case-control	****	*	***	8
**Ristori**	Cohort	****	*	***	8
**Visconti**	Case-control	***	*	***	7
**Yousefi**	Case-control	****	*	***	8

EXCLI Journal 2021;20:1571-1584 – ISSN 1611-2156
Received: February 05, 2021, accepted: October 18, 2021, published: November 19, 2021

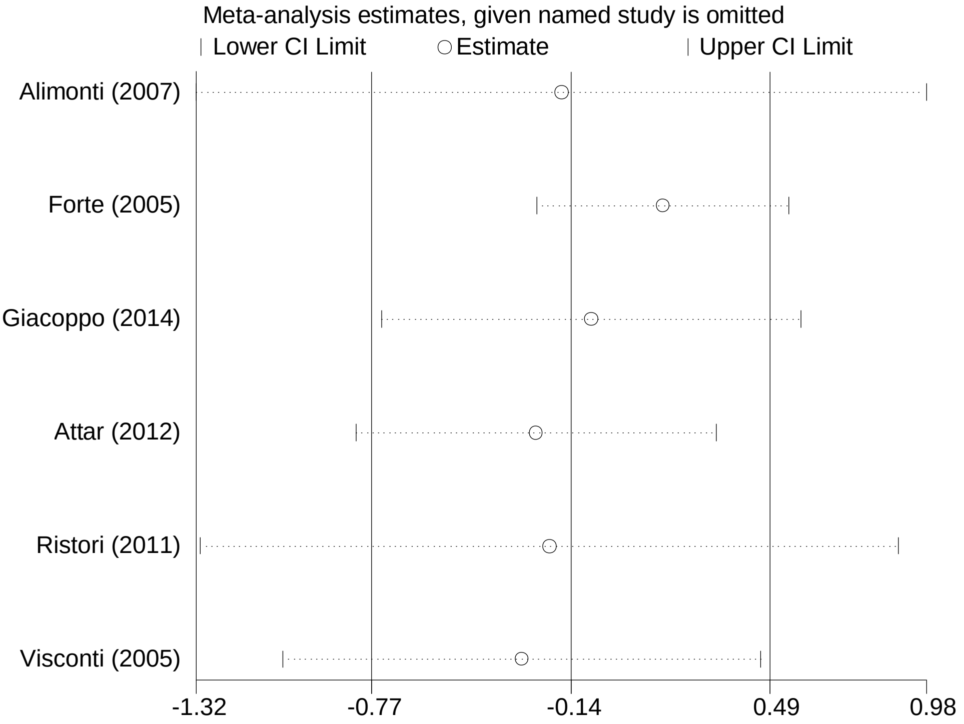

Supplementary Figure 1: Sensitivity analysis findings after excluding each study for mercury (Hg)

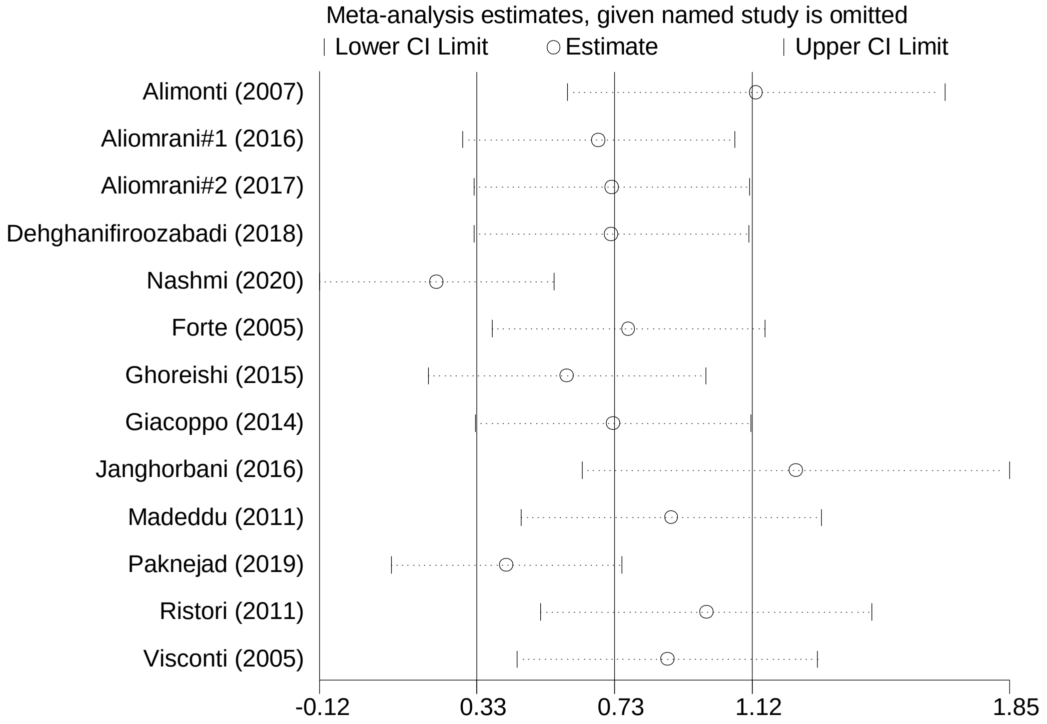

Supplementary Figure 2: Sensitivity analysis findings after excluding each study for lead (Pb)

EXCLI Journal 2021;20:1571-1584 – ISSN 1611-2156
Received: February 05, 2021, accepted: October 18, 2021, published: November 19, 2021

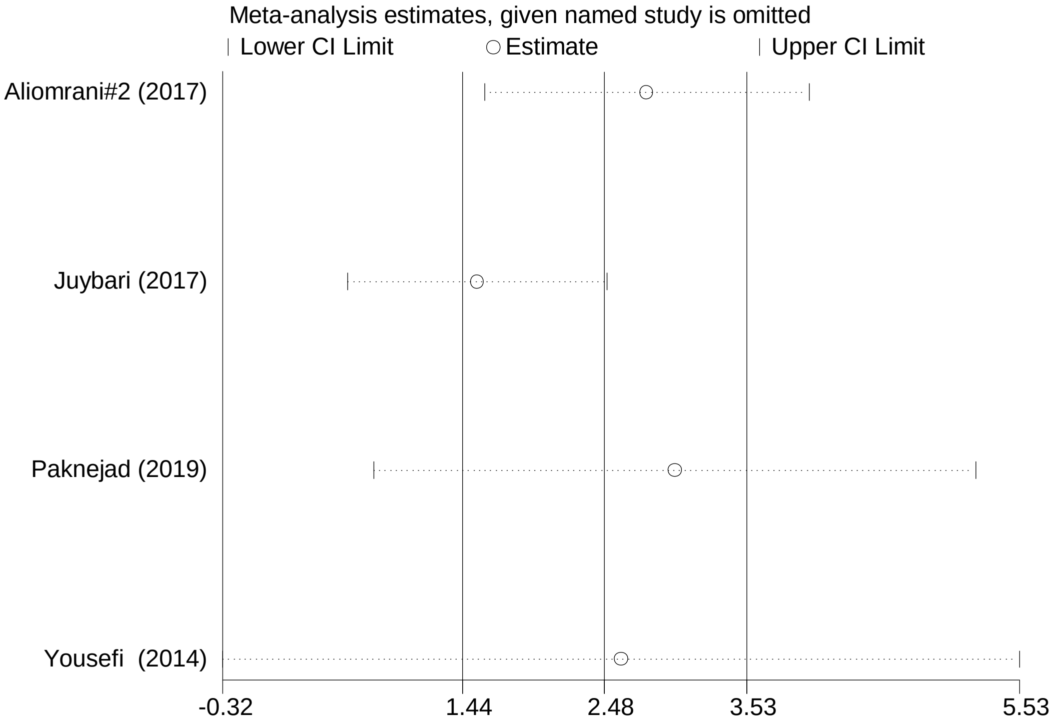

Supplementary Figure 3: Sensitivity analysis findings after excluding each study for Arsenic (As)

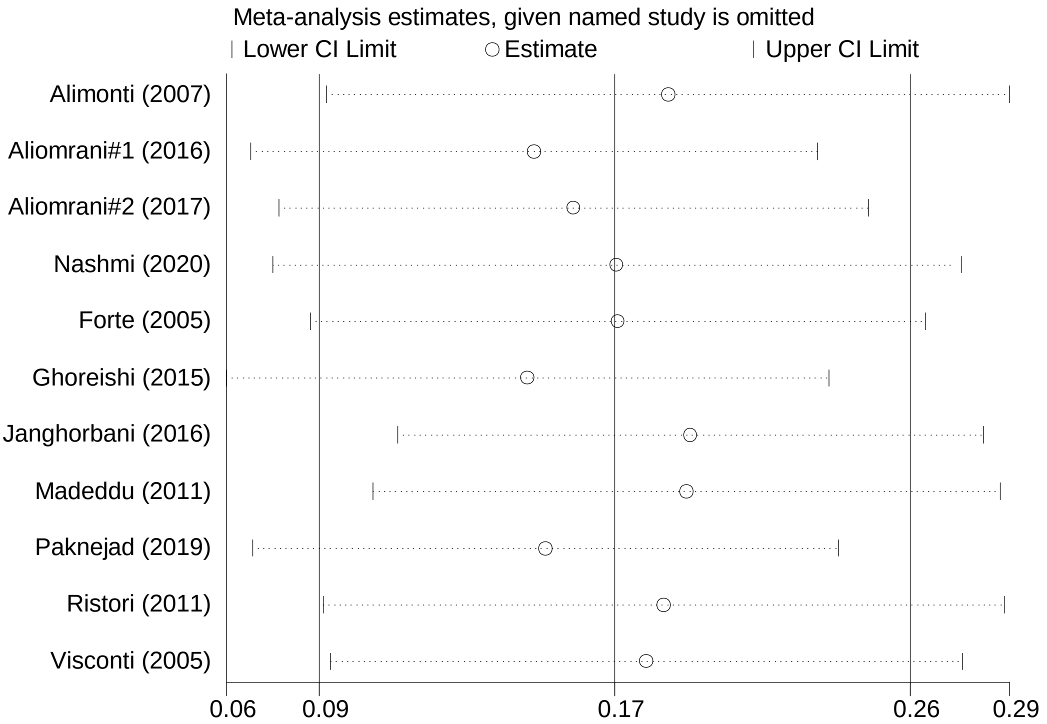

Supplementary Figure 4: Sensitivity analysis findings after excluding each study for Cadmium (Cd)
